# Use of Infrared Thermography in Visualizing Erythema Migrans

**DOI:** 10.7759/cureus.89242

**Published:** 2025-08-02

**Authors:** Natalia I Brothers, Alison Rebman, Jonathan M Zenilman, John Aucott

**Affiliations:** 1 Lyme Disease Research Center, Division of Rheumatology, Department of Medicine, Johns Hopkins University, Baltimore, USA

**Keywords:** borrelia burgdorferi, erythema migrans, inflammation, infrared imaging, lyme disease, skin, thermography

## Abstract

Infrared thermography (IRT) is a non-invasive imaging technology that visualizes heat patterns on the surface of the body. IRT measures deviations from baseline body temperature that correspond to areas of increased peripheral perfusion. The use of IRT in Lyme disease is novel. The present study presents three case examples and explores IRT as a tool for detecting and understanding the erythema migrans (EM) lesions in patients diagnosed with Lyme disease. Examination of these examples revealed these lesions as round or oval areas of increased heat and prominent venous drainage. IRT images are able to identify single and disseminated EM lesions. This study highlights the potential of IRT to improve EM identification.

## Introduction

Lyme disease is a multi-stage, tick-borne disease caused by *Borrelia burgdorferi*, a spirochete transmitted by the Ixodes tick species [1]. The acute stage of Lyme disease is characterized by a localized infection of the skin at the site of a bite by an infected tick. This infection involves cutaneous tissues, triggering an inflammatory cascade by the innate immune system and eliciting cytokine release into the tissue and bloodstream. Several pro-inflammatory markers are upregulated during this local, acute immune response, including IL-1β, IL-6, and TNF-α, which have numerous functions, including increasing vascular perfusion of infected skin [2].

Increased blood flow to the inoculation site promotes rapid migration of immune cells into the tissue, allowing for prompt containment of the infection. This process is initiated by vasodilatation of the surrounding vasculature, mediated by TNF-α [2]. TNF-α, as a pyrogenic cytokine, elicits heat by stimulating the production of prostaglandin E2, which activates thermoregulatory neurons in the hypothalamus [3]. IL-1β stimulates nitric oxide (NO) and prostaglandin E2 (PGE2) production, causing vasodilation and increased vascular permeability, leading to erythema and edema [4]. IL-6 also plays a role in the recruitment of inflammatory cells into the site of infection [5]. These cytokines work in tandem to increase blood flow, heat, and edema, contributing to the diagnostic hallmark of acute Lyme disease, a visible erythema migrans (EM) lesion [2]. While a primary EM occurs at the site of the infected tick bite, secondary lesions may occur through spirochete dissemination from the initial bite location via hematogenous spread [1]. The EM itself may be asymptomatic or accompanied by edema, and occasionally mild pruritus or discomfort may occur. EM also varies by color, shape, and size, which can be challenging for detection and diagnosis by both patients and providers [6]. A constant among primary and secondary EM lesions is the presence of erythema and warmth, which can aid in their identification [1,6]. However, there are no widely available standardized tools for accurately identifying and quantifying these clinical signs.

Fluctuation in body temperature is a recognized indicator of inflammation and infection in clinical practice [7,8]. Changes in heat distribution are regulated by a homeostatic mechanism, known as thermoregulation, that utilizes blood as the primary medium for heat transfer. These heat patterns can be visualized through infrared imaging [9]. Infrared thermography (IRT) is a non-invasive imaging technique for capturing heat emission based on the electromagnetic spectrum [8,9]. The human eye can only perceive color wavelengths within the visible region of this spectrum, spanning from 400 nm to 700 nm [10]. Infrared cameras detect wavelengths that are invisible to the human eye, from 700 nm to 1000 nm, quantify their energy values, and express each value through a range of visible colors [10]. Colors range from white, red, yellow, green, blue, purple, and black and depict a temperature gradient from hottest to coldest [10].

IRT has emerged as a useful tool in oncology, dermatology, and infectious diseases (ID) to assess cancer diagnosis, inflammation, and viral infections. For example, melanoma and non-melanoma skin cancers have shown marked differences in their thermal patterns [11], indicating that IRT could assist with differentiation between skin cancer types [11]. In dermatology practice, IRT has helped clinicians assess allergic dermatitis, acne vulgaris, psoriasis, localized scleroderma, cellulitis, and hidradenitis suppurativa, among other conditions [12-15]. A 2015 study discovered the practicality of IRT in generating a complete visual of acute herpes zoster caused by the varicella-zoster virus; lesion sites were warmer than unaffected areas and denoted localized inflammation [16].

We explored the clinical use of IRT among patients with acute Lyme disease infection and present three illustrative case examples. We then discuss potential applications of this technology for improving EM identification and prompt diagnosis. This report was previously presented as a poster at the Johns Hopkins Bloomberg School of Public Health 3rd Annual Ticks and Tickborne Diseases Symposium on April 30, 2025.

## Case presentation

Study participants

Adult patients with a clinical diagnosis of Lyme disease (≥ 18 years) were physician-referred or recruited from primary and urgent care practices in Maryland from 2010 to 2011 and enrolled in a longitudinal cohort study. Eligible participants had provider-diagnosed EM ≥ 5 cm in diameter, acute illness of < 3 months, and no prior antibiotic treatment for their current Lyme disease episode. This study was conducted in accordance with the standards of the 1964 Declaration of Helsinki. It was approved by the Institutional Review Board of the Johns Hopkins University School of Medicine (approval number: NA_00011170), and written consent was obtained from all study participants prior to the completion of study-related activities. This paper focuses on images and data from three of these participants, who were selected as illustrative examples of the range of EM in early Lyme disease, including single and multiple lesions and lesions with vesicular centers. The three participants received treatment with doxycycline for 21 days at their initial visit.

Infrared camera

Infrared images were obtained using a 7320 Epidermal Thermal Imaging Professional Series (ETI-P) camera system (FLIR Systems, Inc., Wilsonville, Oregon) with a 320 x 240-pixel resolution (Infrared Cameras, Inc., South Range, Minnesota). Imaging contrast was provided by the IR Flash Professional Thermal Image Analysis version 2.10.1.14 software (InfraRed Integrated Systems Ltd., Milton Keynes, United Kingdom) with temperature accuracy of ±1°C. The camera provided an effective operating temperature range of -20°C to 50°C and a spectral range of 7-14 μm. Visible light photographs were taken with a 12-megapixel Nikon Coolpix L22 camera for comparison (Nikon Corporation, Tokyo, Japan).

Infrared and visible light photos were taken in a well-lit, temperature-controlled exam room at initial (at the time of enrollment and antibiotic treatment initiation) and follow-up (three weeks later at the end of treatment) visits. Photos were uploaded into the IR Flash Professional Thermal Imaging Analysis Software, and the EM sites were labeled using the “zone” feature. Once a zone was drawn around the EM, a temperature gradient was visible, and heat signature data were exported into a Microsoft Excel spreadsheet (Microsoft Corp., Redmond, WA) for analysis.

Case 1

**Figure 1:**
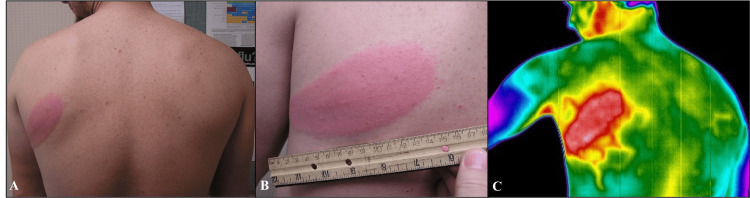
Erythema migrans (EM) of Case 1 at baseline Images were taken using a visible light camera (A, B) and an infrared camera (C) in a room-temperature-controlled clinical exam room. The uniformly red lesion on the left scapula measured at 15 x 7 cm. The red and white regions throughout the elliptical EM ranged from 33.2–33.8°C and 33.9–34.2°C, respectively. Comparatively, temperatures of the surrounding, unaffected tissue ranged from 33.0 to 33.1°C. Several areas of dispersed heat projecting from the oval lesion were present and denoted possible heat leakage through local venous blood vessels.

A previously healthy 28-year-old White man presented with a uniformly red, oval skin lesion on his left scapula after doing yard work several days prior (Figure 1). Fatigue was associated with his rash onset. His EM measured at 15 x 7 cm. IRT captured the elliptical EM as hotter in the center compared to the EM border. Potential heat channels radiating from the lesion were noted. These projections from the EM were thermally similar to the EM itself and were interpreted as evidence of venous drainage from the lesion. At the three-week follow-up visit, the EM was no longer detected by visible light or IRT. Lyme two-tier serology results at the initial and follow-up visits were negative. The initially negative serology is consistent with the fact that patients recently infected with *Borrelia burgdorferi* may have negative serology results, as the serologic assays are only able to detect antibodies that can take several weeks to develop [17]. In addition, those who receive antibiotic treatment early in their disease may be less likely to seroconvert on convalescent serology [17]. According to the Association of Public Health Laboratories (APHL), patients with an EM lesion that is identified by a healthcare provider who lives in or has visited a Lyme-endemic area can be diagnosed with acute Lyme disease even in the absence of positive lab results [18].

**Figure 2:**
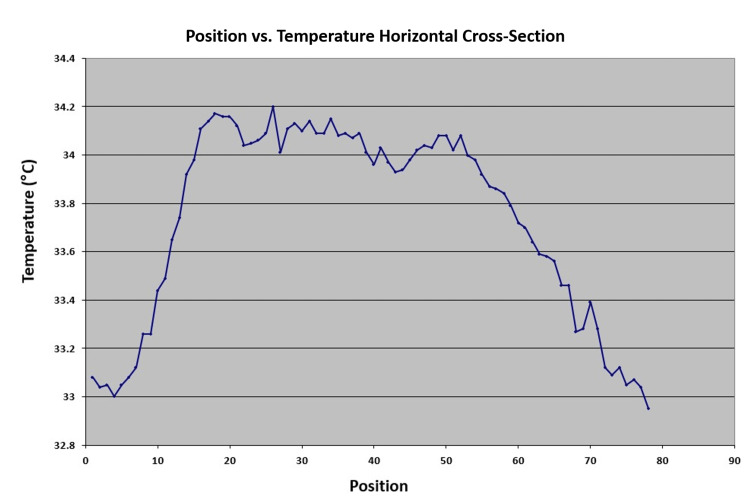
Horizontal cross-section of Case 1's erythema migrans (EM) A horizontal cross-section of the EM lesion was created to serve as a reference for heat values and the corresponding infrared color spectrum. Each point along the X-axis, referred to as a position point, represents evenly spaced pixels in the infrared image that span the horizontal length of the EM lesion. The blue-green region representing unaffected cutaneous tissue measured at 33.0-33.1°C (Figure 1C). The yellow surrounding the EM was measured at 33.1-33.2°C (Figure 1C). The red region measured at 33.2-33.8°C, and the white region measured at the warmest values of 33.9-34.2°C (Figure 1C).

Using the Case 1 EM as a basis, the IR Flash Professional Thermal Imaging Analysis Software recorded a temperature range of 33°C to 34.2°C for uniformly red lesions and 33.2°C to 35°C in the red and white areas corresponding to bull's-eye lesions (Figure 2).

Case 2

**Figure 3:**
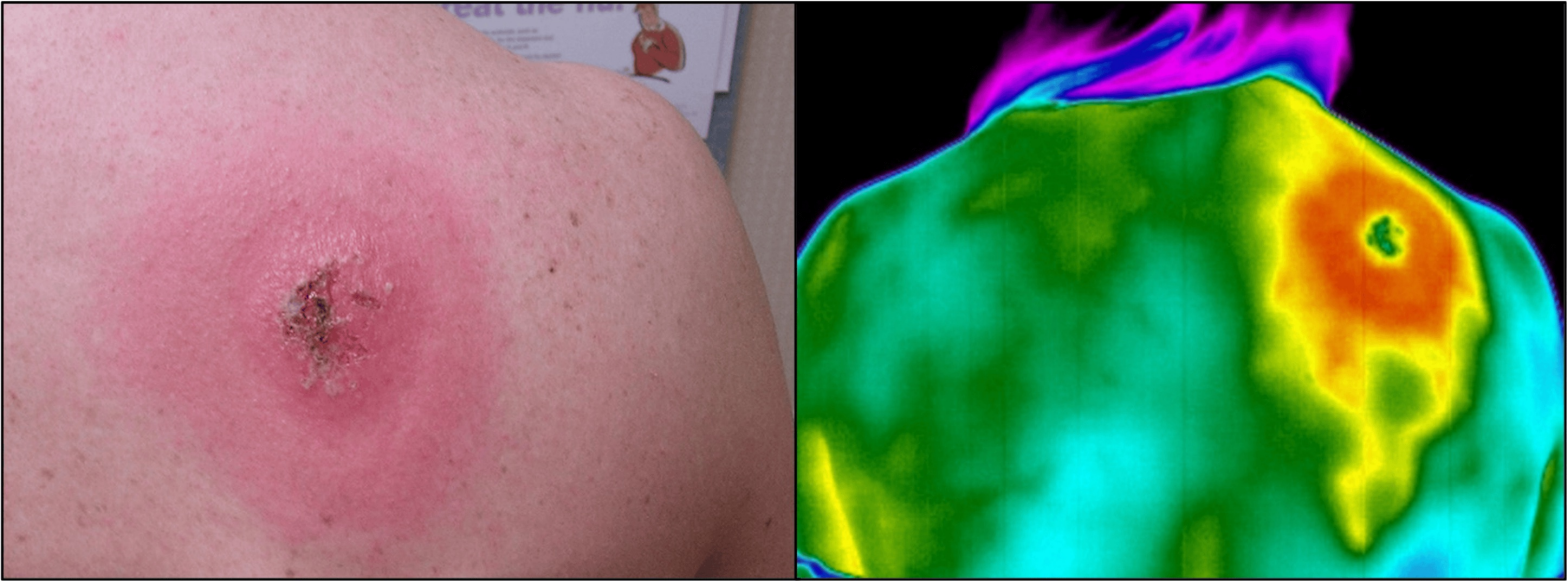
Erythema migrans (EM) of Case 2 at baseline Images were taken with visible light (A) and infrared (B) cameras in a room-temperature-controlled clinical exam room. The EM had a bull’s-eye pattern with evidence of healing bullae, as suggested by the formation of eschar. The lesion measured at 9 x 10 cm on the right scapula. Heat extensions, or channels, are visible by infrared thermography (IRT), as in Figure 1.

**Figure 4:**
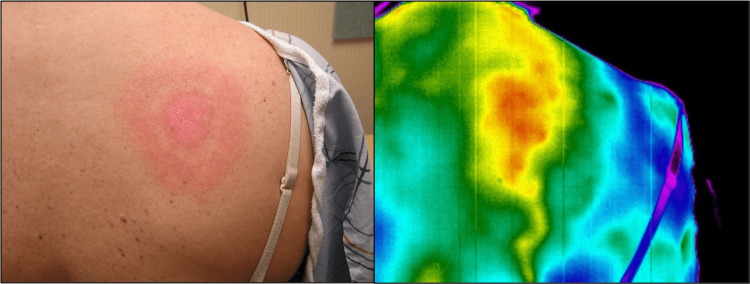
Erythema migrans (EM) of Case 2 at three-week follow-up After a 21-day course of doxycycline treatment, the EM was still visible on routine photography. Though the bullae and eschar were absent, the heat signature from the baseline photos remained.

A 50-year-old White woman presented with a “painful and itchy,” round red skin lesion on her right scapula, muscle and joint pain, paresthesia in her right arm, and a fever (Figure 3). She spent time in her yard five days before the onset of her symptoms. Past medical history included remote breast cancer with a right axillary lymph node resection. Her EM measured at 9 x 10 cm and had a bull’s-eye appearance with central bullae and partially formed eschar. In IRT analysis, the central region of the EM displayed a cooler center compared to the surrounding erythema due to the overlying eschar produced by the infection. Furthermore, the central clearing within the bull’s-eye pattern was slightly less warm than the visibly red areas. As depicted in Case 1, heat projections extended from the EM. The lesion was still visible at the three-week follow-up visit (Figure 4). The heat signature after three weeks was consistent with IRT data from the initial visit. The bullae and eschar healed within the three-week period, and IRT reflected this. Lyme serology at the initial visit was negative, as seen in Case 1. However, at the three-week follow-up, Lyme serology was positive on total enzyme immunoassay (EIA) with a value of 3.06 and two positive bands on IgM immunoblot. These results showed a typical pattern seen in early localized Lyme disease, with development of an IgM response after several weeks of infection [18].

Case 3

**Figure 5:**
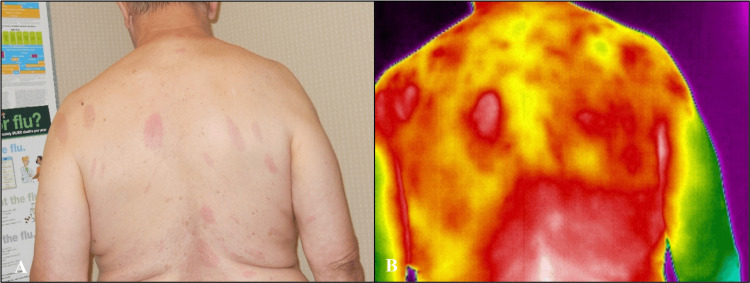
Posterior disseminated erythema migrans (EM) of Case 3 at baseline Images were taken with visible light (A) and infrared (B) cameras in a room-temperature-controlled clinical exam room. Disseminated lesions of various sizes reveal larger areas of heat and inflammation by infrared thermography compared to what is visible to the naked eye.

**Figure 6:**
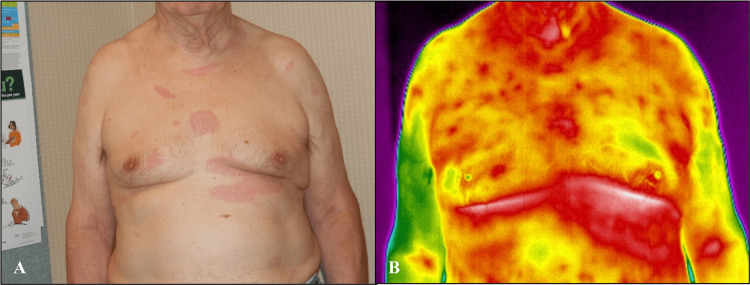
Anterior disseminated erythema migrans (EM) of Case 3 at baseline Photos were captured with visible light (A) and infrared (B) cameras in a room-temperature-controlled clinical exam room. The primary lesion, located on the left breast, is identified in infrared thermography (IRT) as an area of increased heat and inflammation. As with the posterior disseminated lesions, a greater extent of inflammation is exhibited by IRT.

A 68-year-old White man presented with multiple, disseminated EM lesions of various sizes on his anterior and posterior thoracic and lumbar regions (Figure 5, Figure 6). He noticed chills, fatigue, headaches, and a fever eight days before the primary rash appeared while vacationing in the mountains. He noted that the primary rash, located under the left breast, was not painful or itchy. Further symptoms, as the secondary lesions developed, were nausea, vomiting, and low back pain. The largest recorded EM was 15 x 5 cm, and all lesions were red and oval. IRT revealed areas of increased heat and inflammation radiating outward from the EM lesions anteriorly and posteriorly. The disseminated lesions were undetected by visible light or IRT at the three-week follow-up visit, as with Case 1. Case 3 had a positive Lyme two-tier serology result at the initial visit, with three positive IgM bands and an EIA >5.00. This is consistent with a more established immune response associated with disseminated infection; per the Centers for Disease Control and Prevention (CDC) clinical and diagnostic criteria, serologic testing has a 99% specificity and >87% sensitivity for disseminated Lyme disease cases [18].

## Discussion

Accurate identification and timely diagnosis of EM remain a persistent challenge in medicine, with misdiagnosis or delayed treatment resulting in the progression of Lyme disease symptoms and increased risk for disseminated manifestations and post-treatment sequelae [19]. As demonstrated in this study, EM differs in terms of the presence of a bull’s-eye or target-shaped lesion, its elliptical or circular shape, and the number of lesions. This variability and the potential difficulty in seeing some EM under normal light conditions, particularly those on darker skin, emphasize the importance of enhancing identification methods [19]. As the use of IRT in Lyme disease is novel, further studies are needed to explore its application in a larger cohort of patients with a broader variation of EM presentations.

In this study, IRT distinguished inflammation at the infection site from unaffected skin by detecting localized areas of elevated heat that corresponded with the EM location. IRT provided a real-time, non-invasive technique for visualizing heat patterns of one or multiple lesions. In Case 2, IRT illustrated a persistent thermal signature of an EM lesion following a 21-day course of doxycycline, revealing its potential for monitoring inflammation during and after treatment. In all three cases, IRT depicted an unexpected extent of the heat associated with EM compared to what was perceived in visible light. This was seen in infrared images of Cases 1 and 2, which showed heat projections branching from the EM site. We hypothesize that these channels of heat depict venous drainage during acute *Borrelia burgdorferi* infection. One study found that blood vessels become visible in conditions that cause circulatory alterations, supporting our hypothesis [20].

One limitation of our study of IRT is its inability to specifically diagnose an EM rash as Lyme disease, as it can only identify abnormalities in skin temperature. A recent review of IRT in clinical practice highlighted that there are no standardized protocols for IRT, with each protocol varying depending on the camera manufacturer [10]. Additionally, thermoregulation is variable between individuals and affected by factors like fat content and energy metabolism [10]. Individuals with higher fat accumulation tend to have lower body surface temperatures, while females generally exhibit higher surface temperatures than males [10]. These limitations underscore the need for more research on IRT.

Despite its limitations, this study shows that IRT may be useful as an early screening tool for detecting single and multiple erythema migrans lesions with increased skin temperature and inflammation, distinguishing EM in different skin tones, and monitoring inflammation after standard antibiotic treatment. Its use, in conjunction with epidemiological factors and patient-reported symptoms, can support a clinical diagnosis of Lyme disease.

## Conclusions

IRT provides an effective, noninvasive method of visualizing single and disseminated EM lesions. It offers insight into the underlying inflammatory processes of acute *Borrelia burgdorferi *infection. Additional research is required, as the application of IRT in infectious disease literature is limited and previously nonexistent in the context of Lyme disease. IRT can be utilized as a screening tool for diagnosing Lyme disease in cases where a distinctive bull’s-eye rash is absent, or EM is difficult to appreciate under visible light conditions, or in patients with highly pigmented skin.
